# Dataset on brand culture and perceived value of offerings to customers in the hospitality industry in Nigeria

**DOI:** 10.1016/j.dib.2018.04.147

**Published:** 2018-05-05

**Authors:** Joy Dirisu, Rowland Worlu, Adewale Osibanjo, Taiye Borisade, Maxwell Olokundun, Tolu Atolagbe, James Obi

**Affiliations:** Covenant University, Nigeria

## Abstract

This study critically examines the role of brand culture in influencing the perceived value of offerings to customers within the hospitality industry in Nigeria. In today's competitive market, the extent at which organizations disregard the importance of developing a strong brand culture before communicating their value to the outside world has become worrisome. Hence, this study filled in the gaps and a total of 434 customers drawn from six different hotels in Lagos state, Nigeria, were sampled. The data were analysed using Structural Equation Modelling (SEM). Management of these hotels were able to define their expectations in order to deliver a consistent brand experience to their customers. The result showed that brand culture has positive significant influence on the perceived value of offerings to customers. Important recommendations have also been made.

## 1 Introduction

Brands consist of both intangible and tangible elements [1] and as such, it brings to the fore the cultural perspective embedded therein. Brand culture as a concept refers to “the cultural codes developed by brands at a significant level, which influence the comprehension and the value of a brand in the marketplace” [3]. While a lot of extant studies in the developed countries [5], [6], [7] have suggested that brand culture was advantageous to organisations, it also goes to say that the absence of brand culture could affect some critical aspects of modern-day brands and inevitably, the customers’ consumption experience and value. Therefore, this study was aimed at determining the role of brand culture in influencing the perceived value of offerings to customers especially in the hospitality industry.

**Specification Table**

**Table:** 

**Subject area**	Business Management
**More Specific Subject Area:**	Brand Management and HRM
**Type of Data**	Primary data
**How Data was Acquired**	Through questionnaire
**Data format**	Raw, analyzed, Inferential statistical data
**Experimental Factors**	Population consisted of selected Hospitality firms in Nigeria. The researcher-made questionnaire which contained data on brand culture and perceived value of offering to customers were completed
**Experimental features**	Promotion of brand culture is important and is an essential factor of organisational branding in an increasingly competitive environment.
**Data Source Location**	Lagos, Nigeria
**Data Accessibility**	Data is included in this article

**Value of data**

○ The data can be used by managers to properly make decisions that in the long-run would increase their perceived value of offerings to the customers.
○ The data can be used to enlighten managers on the importance of brand culture and how it can be beneficial to increase profitability and customers’ satisfaction.
○ The data provides ample knowledge on how different brand culture can interact effectively by building diverse dimensions of interaction that brings about the creation of a conducive and encouraging organisational climate and culture that affects the way members of an organisation work or function.
○ The data described in this article is made widely accessible to facilitate critical or extended analysis.

## 2 Data

**Fig. 1:**
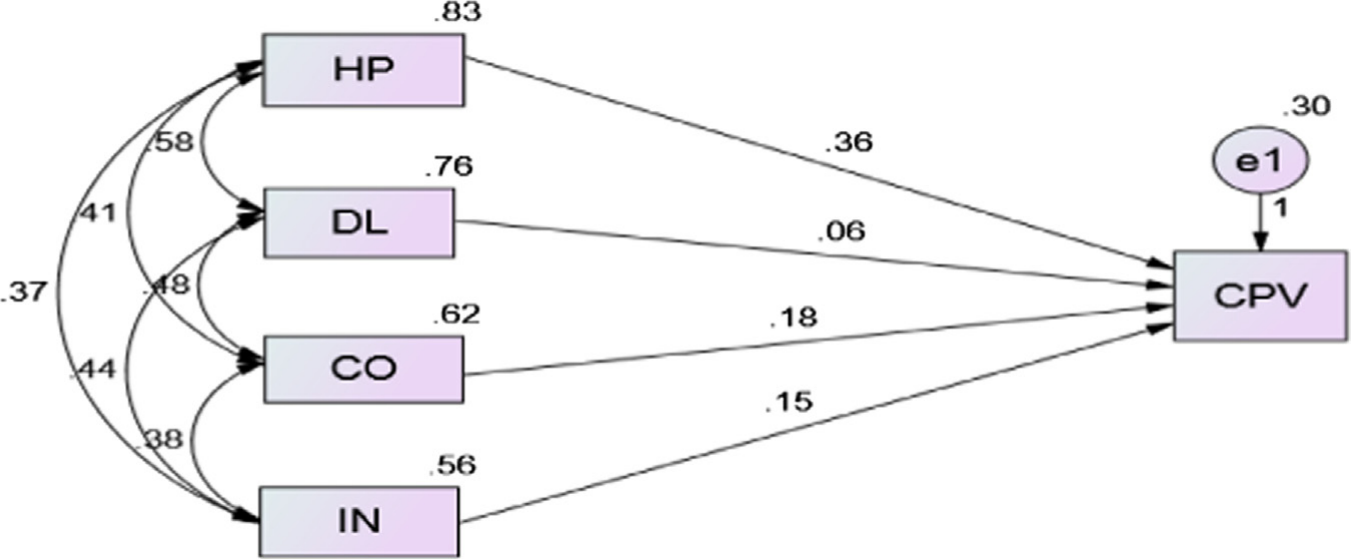
Standardized Regression Weights. Source: Field Survey, 2017.

The data comprised raw inferential statistical data on the effect brand culture on perceived value of offerings to customers of hospitality industry. The study population of this research comprises customers of selected six (6) hotels from a list of 131 hotels adjudged to be the top performing/most popular hotel brands in Lagos State by Tripadvisor (2017). The items in the questionnaire were adopted from brand culture profile (BCP) developed by [2], [5]. While customer perceived values were measured based on some indicators adapted from previous studies by scholars such as [4], [6]. Explicitly, a proposed framework model has been tested using structural equation modelling (SEM) to show the relationship between observed and unobserved variables. A model fit was evaluated by examining several fit indices which include: chi-square (χ2), chi-square/degree of freedom (χ2/df), Goodness-of-Fit Index (GFI), Comparative Fit Index (CFI), Standardized Root Mean Residual (SRMR) and Root Mean Square Error of Approximation (RMSEA). Having run the test, the SEM was obtained, and results of fit indices as presented in Fig. 1 and Table 1, Table 2 respectively.

**Table 1:** The model fit summary showing the goodness of fitness.

Goodness of fit	SEMs value	Recommendation values	Remarks
Chi­Square/Degree of Freedom (CMIN/DF)	2.836	≤3.00	Acceptable fit
Normed Fit Index (NFI)	0.949	≥.90	Good fit
Comparative Fit Index ( CFI)	0.933	≥.90	Good fit
Incremental Fit Index (IFI)	0.987	≥.90	Very Good fit
Root Mean Squared Error of Approximation (RMSEA)	.055	≤.08	Good fit
Goodness of Fit (GFI)	.963	≥.90	Good fit

**Table 2:** Regression weights.

DV		IV	Estimate	S.E.	C.R.	P	Label
CPV	<---	HP	.361	.042	8.529	***	Sig
CPV	<---	DL	.056	.053	1.057	.001	Sig
CPV	<---	CO	.176	.050	3.529	***	Sig
CPV	<---	IN	.148	.051	2.917	.004	Sig

The value of RMSEA is 0.055, which is considered satisfactory (less than 0.08). On top of that, the incremental fit, NFI, TLI, CFI, and GFI were above 0.90 as suggested by [2].

Before conducting the analysis to infer the hypotheses, the data were already tested for linearity, normality, homoscedasticity and multi-collinearity. All these basic assumptions were acceptable and prove that the data meet the conditions of basic assumption in regression analysis [2].

## 3 Experimental design, materials and methods

The data presented was based on a quantitative study. A descriptive research design was adopted in this study to obtain the opinions of customers on their understanding of brand culture of the selected hotels and the extent to which it influences customers’ perceived value.

Survey method was considered appropriate as data collection method based on the fact that it allows for the collection of standardized data that permits the researcher to produce information for answering the how, who, what and when questions regarding the subject matter. Customers of The Wheatbaker Hotel, Ikoyi; Southern Sun Hotel, Ikoyi; Radisson Blu Anchorage Hotel, Victoria Island; Royal View Hotel and Suites, Mafoluku-Oshodi; Westland Hotels and Suites, Ikotun and West View Hotel, Mafoluku-Oshodi were selected for the study.

The use of primary source of data (questionnaire) was used for collecting data from a cross section of customers across sample hotels. The study employed a combination of structured and unstructured question items. The collected data were coded and entered into SPSS version 22. Data analysis was done; using Statistical Package for Social Sciences-22. Although Statistical Package for Social Sciences may be limited when it comes to advanced modeling and development of statistical approaches.

Statistical Package for Social Sciences makes in-depth data analysis quicker because the programme knows the location of the cases and variables. It also comes with more procedures of screening the information in preparation for further analysis. More importantly, Statistical Package for Social Sciences is designed to make certain that the output is kept separate from the data itself particularly because it stores all results in a separate file that is different from the data. Data was analyzed using inferential statistical tests which involved structural equation modelling (SEM).

## 4 Ethical considerations

The researchers ensured that respondents were well informed about the background and the purpose of this research and they were kept abreast with the participation process. Respondents were offered the opportunity to stay anonymous and their responses were treated confidentially.

## 5 Academic and managerial implications

This study revealed that brand culture has significant influence on the perceived value of offerings to customers within the hospitality industry. The requisite for hospitality industry to develop different elements useful in the identification and differentiation of service as complex as the environments in which they operate becomes necessary on a continual basis. Some of these elements include: logos, names, designs, icons, norms, cultural traits and phenomena embedded inside the elements, as well as cultural traditions, perceptions and emotional characteristics which these cultural traits represent. Hence, this present study has extensive implications for the hospitality sector, managers, government, researchers and undergraduate students in this regard. To this end, the data presented in this article is imperative for more comprehensive analysis or investigation.
